# Supplementary material for: Seroepidemiology of Toxoplasma gondii infection in people with alcohol consumption in Durango, Mexico
Source: PLoS One. 2021 Jan 28;16(1):e0245701. doi: 10.1371/journal.pone.0245701 (PMC7842906; doi:10.1371/journal.pone.0245701)
Supplement: S1 File — (DOCX) [file pone.0245701.s001.docx]

**CUESTIONARIO-*T. g.***

Fecha: Folio:

Nombre:

Institución:

**Datos sociodemográficos:**

1) Edad:

2) Sexo: (1) Femenino (2) Masculino

3) Lugar de nacimiento:

1. Durango, México
2. Otro Estado de México. ¿Cuál?
3. Otro país. ¿Cuál?

4) Lugar de residencia:

1. Durango
2. Otro Estado de México. ¿Dónde?
3. En el extranjero. ¿Dónde?

5) Área de residencia:

1. Urbana (2) Suburbana (3) Rural

6) Escolaridad:

(1) No estudios (0 años)

(2) Primaria (1-6 años)

(3) Secundaria o preparatoria (7-12 años)

(4) Profesional o posgrado (13 o más años)

7) Ocupación:

| (1) Agricultura  (2) Ama de casa  (3) Comerciante  (4) Construcción | (5) Empleado(a)  (6) Estudiante  (7) Ganadería  (8) Jornalero(a) | (9) Obrero(a)  (10) Profesionista | (11) Ninguna  (12) Otra ¿Cuál? |
| --- | --- | --- | --- |

8) Nivel socioeconómico: (1) Bajo (2) Medio (3) Alto

**Datos clínicos:**

9) Estado de salud:

1. Sano (a) (2) Enfermo (a) ¿De qué?

10) ¿Ha tenido alguna vez ganglios inflamados en su cuello o nuca o alguna otra parte de su cuerpo? (1) Sí (2) No

11) ¿Sufre frecuentemente de dolor abdominal? (1) Sí (2) No

12) ¿Sufre frecuentemente de dolores de cabeza? (1) Sí (2) No

13) ¿Tiene problema de su memoria? (1) Sí (2) No

14) ¿Sufre de mareos? (1) Sí (2) No

15) ¿Tiene problema con sus reflejos? (1) Sí (2) No

16) ¿Escucha bien? (1) Sí (2) No

17) ¿Ve bien? (1) Sí (2) No

18) ¿Ha recibido alguna transfusión sanguínea? (1) Sí (2) No

19) ¿Ha recibido algún trasplante? (1) Sí ¿Qué tipo? (2) No

20) ¿Ha padecido hepatitis? (1) Sí (2) No

21) ¿La(o) han operado alguna vez?

(1) Sí ¿De qué? (2) No

**Preguntas para mujeres**

22) Número de embarazos: _______

23) Número de partos: _______

24) Número de cesáreas: _______

25) Número de abortos: _______

26) Número de mortinatos _______

**Datos epidemiológicos:**

27) ¿Ha tenido gatos en su casa? (1) Sí (2) No

28) ¿Hay gatos en casas cercanas a la suya? (1) Sí (2) No

29) ¿Limpia usted los excrementos de gatos? (1) Sí (2) No

30) ¿Ha tenido perros en su casa? (1) Sí (2) No

31) ¿Ha tenido pajaritos en su casa? (1) Sí (2) No

32) ¿Ha criado animales de granja o de otro tipo? (1) Sí ¿Cuáles? (2) No

33) ¿Ha viajado fuera del país? (1) Sí ¿a dónde? (2) No

34) ¿Ha viajado a otros estados de México? (1) Sí ¿a dónde? (2) No

35) ¿Consume carne de puerco? (1) Sí (2) No

36) ¿Consume carne de res? (1) Sí (2) No

37) ¿Consume carne de cabra? (1) Sí (2) No

38) ¿Consume carne de borrego? (1) Sí (2) No

39) ¿Consume carne de jabalí? (1) Sí (2) No

40) ¿Consume carne de pollo? (1) Sí (2) No

41) ¿Consume carne de guajolote (pavo)? (1) Sí (2) No

42) ¿Consume carne de paloma? (1) Sí (2) No

43) ¿Consume carne de pato? (1) Sí (2) No

44) ¿Consume carne de codorniz? (1) Sí (2) No

45) ¿Consume carne de conejo? (1) Sí (2) No

46) ¿Consume carne de venado? (1) Sí (2) No

47) ¿Consume carne de ardilla? (1) Sí (2) No

48) ¿Consume carne de caballo? (1) Sí (2) No

49) ¿Consume carne de tlacuache? (1) Sí (2) No

50) ¿Consume carne de armadillo? (1) Sí (2) No

51) ¿Consume carne de iguana? (1) Sí (2) No

52) ¿Consume carne de víbora? (1) Sí (2) No

53) ¿Consume carne de pescado y mariscos? (1) Sí (2) No

54) ¿Consume carne de algún otro animal (rata, zorrillo, avestruz, etc.)?

(1) Sí ¿Cuál(es)? (2) No

55) ¿Cuantos días a la semana acostumbra a comer carne (de cualquier tipo)?

(1) Nunca (2) Tres días o menos (3) de 4 a 7 días

56) En caso de consumir carne (de cualquier tipo) ¿Cómo acostumbra a comerla?

(1) Cruda (2) Poco cocida (3) Bien cocida.

57) ¿Ha consumido carne seca cruda? (1) Sí ¿de qué? (2) No

58) ¿Consume embutidos (salchicha, jamón, salami, etc.)?

(1) Sí (2) No

59) ¿Consume chorizo? (1) Sí (2) No

60) ¿Ha consumido sesos? (1) Sí ¿de qué animal? (2) No

61) ¿Ha consumido hígado? (1) Sí ¿de qué animal? (2) No

62) ¿Consume leche bronca (de vaca, cabra, borrega, o burra)?

(1) Si ¿de qué animal? (2) No

63) ¿Consume verduras crudas sin lavar? (1) Sí (2) No

64) ¿Consume frutas crudas sin lavar? (1) Sí (2) No

65) ¿Consume agua no hervida o no tratada? (1) Sí (2) No

66) ¿Con que frecuencia come fuera de su casa (en restaurantes, puestos, etc.)?

(1) Nunca. (2) De 1 a 10 veces al año. (3) Más de 10 veces al año.

67) ¿Tiene contacto con tierra (excavaciones, jardinería, agricultura, etc.)?

(1) Sí (2) No

68) ¿Acostumbra a lavarse las manos antes de comer? (1) Sí (2) No

69) ¿Toma alcohol (por lo menos una bebida al mes en los últimos 6 meses)?

(1) Sí (2) No

70) ¿Fuma tabaco?

(1) Sí (2) No

71) ¿Consume drogas?

(1) Sí (2) No

72) ¿Tiene promiscuidad sexual?

(1) Sí (2) No

73) ¿De qué material es el piso dentro de su casa?

(1) Recubrimiento (loseta, madera, etc.) (2) Cemento (3) Tierra

74) Agua potable:

(1) Intradomiciliaria (2) Dentro del vecindario o terreno (3) Hidrante público.

75) Eliminación de excretas:

(1) drenaje (2) Letrina, pozo negro, fecalismo, etc.

76) Nivel de hacinamiento (número de personas entre el número de cuartos):

1. No hacinado (hasta 1.5) (2) Semihacinado (1.6-3.5) (3) Hacinado (3.6 y más)

77) Nivel de escolaridad del jefe de la familia:

(1) 7 años o más (2) 4 a 6 años (3) Hasta 3 años
