# Supplementary material for: Seroepidemiology of Toxoplasma gondii infection in people with alcohol consumption in Durango, Mexico
Source: PLoS One. 2021 Jan 28;16(1):e0245701. doi: 10.1371/journal.pone.0245701 (PMC7842906; doi:10.1371/journal.pone.0245701)
Supplement: S2 File — (DOCX) [file pone.0245701.s002.docx]

**QUESTIONNAIRE *T. g.***

Date: Number:

Name:

Institution:

**Sociodemographic characteristics**

1) Age:

2) Sex: (1) Female (2) Male

3) Birthplace:

1. Durango.
2. Another State in Mexico. Where?
3. Abroad. Where?

4) Residence place:

1. Durango.
2. Another State in Mexico. Where?
3. Abroad. Where?

5) Residence area:

1. Urban (2) Suburban (3) Rural

6) Education:

1. No education (0 years).
2. Primary school (1-6 years).
3. Up to high school (7-12 years).
4. College or higher (13 or more years).

7) Occupation

| (1) Agriculture  (2) Housewife  (3) Busyness  (4) Construction | (5) Employee  (6) Student  (7) Cattle raising  (8) Day laborer | (9) Factory worker  (10) Professional | (11) None  (12) Other Which? |
| --- | --- | --- | --- |

8) Socioeconomic level: (1) Low. (2) Medium. (3) High.

**Clinical characteristics**

9) Health status. (1) Healthy (2) Ill. What disease?

10) Have you ever had any lymph node enlargement in neck, nape, or another place in your body?

(1) Yes. (2) No.

11) Do you suffer from frequent abdominal pain?

(1) Yes. (2) No.

12) Do you suffer from frequent headache?

(1) Yes. (2) No.

13) Do you have memory impairment? (1) Yes (2) No

14) Do you suffer from dizziness?

(1) Yes. (2) No.

15) Do you have reflexes impairment? (1) Yes (2) No

16) Do you have hearing impairment? (1) No (2) Yes

17) Do you have vision impairment? (1) No (2) Yes

18) Have you ever received any blood transfusion? (1) Yes. (2) No.

19) Have you ever received any organ transplantation?

(1) Yes. What type? (2) No.

20) Have you ever had hepatitis?

(1) Yes. (2) No.

21) Have you ever had a surgical procedure?

(1) Yes. What type? (2) No.

**Questions for women:**

22) Number of pregnancies:

23) Number of deliveries:

24) Number of cesarean sections:

25) Number of miscarriages:

26) Number of stillbirths:

**Behavioral characteristics**

27) Have you ever had cats at home? (1) Yes. (2) No.

28) Are there cats in houses of the neighborhood?

(1) Yes. (2) No.

29) Do you clean cat feces?

(1) Yes. (2) No.

30) Have you ever had dogs at home?

(1) Yes. (2) No.

31) Have you ever had birds at home?

(1) Yes. (2) No.

32) Have you ever raised farm animals or another type of animals?

(1) Yes. Which? (2) No.

33) Have you ever traveled abroad? (1) Yes. Where? (2) No.

34) Have you ever traveled to Mexican states? (1) Yes. Where? (2) No.

35) Do you eat pork? (1) Yes. (2) No.

36) Do you eat beef? (1) Yes. (2) No.

37) Do you eat goat meat? (1) Yes. (2) No.

38) Do you eat sheep meat? (1) Yes. (2) No.

39) Do you eat boar meat? (1) Yes. (2) No.

40) Do you eat chicken meat? (1) Yes. (2) No.

41) Do you eat turkey meat? (1) Yes. (2) No.

42) Do you eat pigeon meat? (1) Yes. (2) No.

43) Do you eat duck meat? (1) Yes. (2) No.

44) Do you eat quail meat? (1) Yes. (2) No.

45) Do you eat rabbit meat? (1) Yes. (2) No.

46) Do you eat venison? (1) Yes. (2) No.

47) Do you eat squirrel meat? (1) Yes. (2) No.

48) Do you eat horse meat? (1) Yes. (2) No.

49) Do you eat opossum meat? (1) Yes. (2) No.

50) Do you eat armadillo meat? (1) Yes. (2) No.

51) Do you eat iguana meat? (1) Yes. (2) No.

52) Do you eat snake meat? (1) Yes. (2) No.

53) Do you eat fish meat or sea food? (1) Yes. (2) No.

54) Do you eat meat from another animal? (1) Yes. Which? (2) No.

55) How often in a week do you eat meat (of any type)?

(1) Never (2) 3 days or less (3) 4-7 days

56) How cooked do you eat meat?

(1) Raw (2) Undercooked (3) Well done.

57) Have you ever eaten raw dried meat?

(1) Yes. From which animal? (2) No.

58) Do you eat cured meat (sausages, ham, salami, etc.?

(1) Yes. (2) No.

59) Do you eat chorizo? (1) Yes. (2) No.

60) Have you ever eaten animal brains?

(1) Yes. From which animal? (2) No.

61) Have you ever eaten liver?

(1) Yes. From which animal? (2) No.

62) Do you drink unpasteurized milk (from cow, goat, sheep, or donkey?

(1) Yes. From which animal? (2) No.

63) Do you eat unwashed raw vegetables? (1) Yes. (2) No.

64) Do you eat unwashed raw fruits? (1) Yes. (2) No.

65) Do you drink untreated water? (1) Yes. (2) No.

66) How often do you eat out of home (in restaurants, fast food outlets, etc.)?

(1) Never (2) 1-10 times a year (3) >10 times a year

67) Do you have contact with soil (gardening, agriculture, etc.)?

(1) Yes. (2) No.

68) Do you usually wash your hands before eating?

(1) Yes. (2) No.

69) Do you drink alcohol (at least one drink a month in the previous six months)?

(1) Yes. (2) No.

70) Do you smoke tobacco? (1) Yes. (2) No.

71) Do you use drugs? (1) Yes. (2) No.

72) Do you have sexual promiscuity? (1) Yes. (2) No.

73) Type of flooring at home:

(1) Coating (ceramic, wood, etc.)

(2) Concrete

(3) Soil

74) Availability of potable water:

(1) Within the house

(2) In the land

(3) At the street

75) Form of elimination of excreta:

(1) Drainage

(2) Latrine, open air, etc.

76) Crowding: (number of people divided by the number of bedrooms)

(1) No crowding (up to 1.5)

(2) Semi-crowded (1.6 to 3.5)

(3) Crowded (3.6 or more)

77) Education of the head of the family:

(1) 7 years or more

(2) 4 to 6 years

(3) Up to 3 years
