# Supplementary material for: Seroepidemiology of Toxoplasma gondii infection in people with alcohol consumption in Durango, Mexico
Source: PLoS One. 2021 Jan 28;16(1):e0245701. doi: 10.1371/journal.pone.0245701 (PMC7842906; doi:10.1371/journal.pone.0245701)
Supplement: Attachment — Submitted filename: Response to Reviewers.docx [file pone.0245701.s003.docx]

Durango, Dgo. Mexico. December 14, 2020.

Dear Editor,

Please find attached a revised version of our manuscript that has been modified according to the reviewers’ comments. In addition, please find below our response to each of the reviewers’ comments on a point-by-point basis.

We appreciate the valuable comments of the reviewers and we hope the revised manuscript may have more success for publication in the journal *Plos One*.

Kind regards,

Dr. Cosme Alvarado-Esquivel.

Laboratorio de Investigación Biomédica

Facultad de Medicina y Nutrición

Avenida Universidad S/N.

34000 Durango, Dgo. Mexico.

Tel/Fax.: 0052 618 8 271200

Email: alvaradocosme@yahoo.com

**RESPONSE TO THE REVIEWERS’ COMMENTS**

1. Please ensure that your manuscript meets PLOS ONE's style requirements, including those for file naming.

**The manuscript was modified according to the PLOS ONE’s style requirements.**

2.We suggest you thoroughly copyedit your manuscript for language usage, spelling, and grammar. If you do not know anyone who can help you do this, you may wish to consider employing a professional scientific editing service.

**English was revised. The name of the American who review the manuscript was written in the Acknowledgements section. Changes were highlighted in the manuscript. A file (“Supporting information”) with the changes marked in green color was included. A clean copy of the edited manuscript (“Manuscript”) was included.**

3. Please include additional information regarding the survey or questionnaire used in the study and ensure that you have provided sufficient details that others could replicate the analyses. For instance, if you developed a questionnaire as part of this study and it is not under a copyright more restrictive than CC-BY, please include a copy, in both the original language and English, as Supporting Information.

**A questionnaire was included in both the original language and English.**

4. In the Methods, please discuss whether and how the questionnaire was validated and/or pre-tested. If this did not occur, please provide the rationale for not doing so.

**There was no need to perform a validation of the questionnaire because it has been used in a number of epidemiological studies in the same language in the same country. The questionnaire was not used as an evaluation scale but for recording simple variables as age, gender, etc. Items of housing variables were previously validated. This information was added to the Methods section (lines 108-113).**

5. Please clearly define alcohol consumption and specifically outline the criteria that were used to determine consumption. The statement "...at least one drink a month..." is non-specific as different drinks have varying concentrations of alcohol.

**Further information about the definition of alcohol consumption was added to the Methods section (lines 91-96).**

6. In statistical methods, please clarify whether you corrected for multiple comparisons.

**No analysis of groups within individual variables (multiple comparisons) was performed (lines 163-165).**

**Thank you for your valuable comments for improving our manuscript.**

Reviewers' comments:

Reviewer's Responses to Questions

Comments to the Author

1. Is the manuscript technically sound, and do the data support the conclusions?

The manuscript must describe a technically sound piece of scientific research with data that supports the conclusions. Experiments must have been conducted rigorously, with appropriate controls, replication, and sample sizes. The conclusions must be drawn appropriately based on the data presented.

Reviewer #1: No

Reviewer #2: Partly

2. Has the statistical analysis been performed appropriately and rigorously?

Reviewer #1: Yes

Reviewer #2: Yes

3. Have the authors made all data underlying the findings in their manuscript fully available?

The PLOS Data policy requires authors to make all data underlying the findings described in their manuscript fully available without restriction, with rare exception (please refer to the Data Availability Statement in the manuscript PDF file). The data should be provided as part of the manuscript or its supporting information, or deposited to a public repository. For example, in addition to summary statistics, the data points behind means, medians and variance measures should be available. If there are restrictions on publicly sharing data—e.g. participant privacy or use of data from a third party—those must be specified.

Reviewer #1: Yes

Reviewer #2: No

4. Is the manuscript presented in an intelligible fashion and written in standard English?

PLOS ONE does not copyedit accepted manuscripts, so the language in submitted articles must be clear, correct, and unambiguous. Any typographical or grammatical errors should be corrected at revision, so please note any specific errors here.

Reviewer #1: Yes

Reviewer #2: Yes

5. Review Comments to the Author

Reviewer #1:

1. Add the location of study in the title

**The location of the study was added to the Title.**

2. Keywords must be different from title words.

**Changed. All keywords are now different from the Title words.**

3. The rational of the study is not clearly understood.

**The rationale section was rewritten to make it clearer (lines 68-80).**

4. About the inclusion criteria, what about other criteria such as immune deficiency diseases, chronic diseases, etc?????

**The presence of any disease was not a restrictive criterium for inclusion (lines 96-98).**

5. What about the exclusion criteria?

**Exclusion criteria were added (lines 98-99).**

6. The main disadvantage of this study is the lack of a control group.

**This is not a case control study; it was not aimed to determine an association between *T. gondii* infection and alcohol consumption. This study is a cross-sectional study aimed to determine the prevalence of and factors associated with *T. gondii* infection in people with alcohol consumption.**

7. Considering serological tests, what about the range of equivocal results and index values for IgG and IgM?

**There were no equivocal results. Information about index values for IgG and IgM was added (lines 137-140).**

8. Considering low specificity of IgM and IgG in the ELISA test, it is better that the authors perform the high level tests such as Elecsys Toxo IgM assay, PCR assay, …..

**This study was aimed to determine the seroepidemiology of *T. gondii* infection and this aim can only be reached by determining the anti-*T. gondii* IgG and IgM antibodies. These infection markers are well accepted markers used for epidemiological studies.**

9. Add ethical statement number

**Added (lines 170-171).**

**Thank you for your valuable comments for improving our manuscript.**

Reviewer #2:

1- Authors might need to give a bit of context for the study settings including if it was conducted in Durango state or Durango city. It was not clear to the reviewer what area does this study cover. It was not clear as well where the recruitment of participant in the study was conducted (community, health facilities, ….???).

**Information about the study settings was added (lines 99-102).**

2- Would you explain the number mentioned in line 134 (336,606) as the size of population from which the sample was selected?

**An explanation about the size of population from which the sample was selected was added (lines 153-158).**

3- What was the sampling technique employed in this study? How the participants in the study were selected and enrolled?

**Information about sampling was added (lines 101-103).**

4- Would you please highlight if the study duration of 4 years (2014 – 2018) would impact the interpretation of the results?

**Information about a lack of impact of the duration of the study on the interpretations of the results was added to the Discussion section (lines 246-249).**

5- In table 5, the authors presented the logistic regression results of high IgG and some characteristics. The bivariate analysis of this characteristics was not reported in the manuscript.

**Information about results of bivariate analysis of high IgG antibody levels and the characteristics of participants was added (lines 211-219).**

6- The authors didn’t consider a P value of exactly 0.05 as significant, which is debatable.

**We used a *P*<0.05 as statistically significant just to be more stringent in determining associations between *T. gondii* infection and other variables.**

7- In line 172, the authors stated that (crowding was not associated with seropositivity). However, it was mentioned that crowding was associated with (high) antibodies. The authors need to define the term (seropositivity) and make clear distinction between IgG, IgM and both IgG/IgM positive cases. Moreover, the clinical and statistical significance of the levels of IgG need to be explained.

**An explanation about the terms seropositivity, serointensity and high antibody levels were added (lines 141-146).**

8- It was mentioned in line 111 and 112 that “traveling, frequency of eating away from home (in restaurants or fast food outlets)” were studied as an associated factors. In the results section in lines 184 to 188, authors reported that these factors were not found to be associated with seropositivity. However, the reviewer failed to find these factors among the results tables.

**More information about “traveling” was added (line 120). The words “traveled abroad” were included in the Tables. The words “domestic travel” are mentioned in line 203.**

**The variable “frequency of eating away from home” is mentioned in line 205.**

9- In lines 173, 174 and 175, the authors mentioned that visual impairment, history of surgery, and hepatitis were associated with IgG seropositivity. This should be corrected to (negatively associated) as mentioned in line 36.

**Corrected (lines 192-193).**

10- In line 33, the sentence starting (fitty-five …..) is confusing. Please consider mention first that 167 of the IgG positives were also tested for IgM and 55 of them were positive.

**The sentence was modified (lines 32-34).**

11- In line 29, please replace the word (detected) by another word (e.g. studies, measured) to avoid confusion.

**Changed by “searched” (lines 27-29).**

12- Please consider how this study will inform the prevention and control measures.

**Added (lines 288-291).**

**Thank you for your valuable comments for improving our manuscript.**
